# Sense of coherence is associated with the presence of healthy lifestyles in the migrant population

**DOI:** 10.3389/fpsyg.2025.1543830

**Published:** 2025-03-12

**Authors:** Alfonso Urzúa, Diego Henríquez, Alejandra Caqueo-Urízar, Tiare Salazar-Fernández, María Fernanda Esquivel Rojas, Rocío Díaz Vera, Fernanda Hoppe Chacón

**Affiliations:** ^1^Escuela de Psicologìa, Universidad Catòlica del Norte, Antofagasta, Chile; ^2^Centro de Justicia Educacional (CJE), Pontificia Universidad Católica de Chile, Santiago, Chile; ^3^Instituto de Alta Investigación, Universidad de Tarapacá, Arica, Chile

**Keywords:** migration, health, sense of coherence, healthy lifestyle, immigrant

## Abstract

**Background:**

Despite the evidence of the sense of coherence (SOC) as a key factor in stress management and health promotion, there is little information about its connection with healthy lifestyles in migrant population.

**Objective:**

To investigate the relationship between the variables SOC and Healthy Lifestyles in a south-south migrant population.

**Study design:**

Non-experimental, cross-sectional and correlational study.

**Method:**

A total of 1,844 migrants living in Chile participated, selected through a non-probabilistic intentional sampling. The SOC and lifestyle were assessed by applying the FANTASTICO and SOC-13 instruments.

**Results:**

SOC was positively correlated with lifestyle in the migrant population. Sex differences in SOC were identified, suggesting that men have greater access to resources. Age was also positively correlated with SOC and healthy lifestyle. Overall, the importance of considering the particularities of each group in health promotion in migration contexts is emphasized.

**Conclusions:**

This research supports the hypothesis that a strong SOC is positively related to a healthy lifestyle in migrants. The differences found could be explained by sociocultural resources and norms, suggesting the importance of comprehensively addressing aspects related to the mental and physical health of the migrant population, especially in complex migratory contexts.

## 1 Introduction

As of 2020, there were ~281 million international migrants in the world, equivalent to 3.6% of the world population (IOM, 2024). During 2019, in South America, 1.41 million residence authorizations were issued to regular immigrants who settled in Latin American and Caribbean nations (Inter-American Development Bank, 2021). In Chile, as of 2022, the resident foreign population was ~9.3% of the total population (INE, 2022).

The migratory context can expose people to conditions and situations that can affect their physical and mental wellbeing (Rubio León, 2020; Urzúa et al., 2021a, 2023). The relationship between migration and health is complex and its impact can vary significantly among different migrant groups and even among individuals within those groups. There is evidence that both the health and wellbeing of people who migrate are related to pre- and post-migration factors that can positively or negatively affect their health (Rubio León, 2020; Bhugra, 2004; Jurado et al., 2017; Urzúa et al., 2022), as they can, for example, increase vulnerability to disease, increase the migrant's risk behaviors, or, on the contrary, promote good health (Hun and Urzúa, 2023). Examples of factors affecting the health of the migrating population can be found in reviews on Xenophobia (Suleman et al., 2018) or acculturation strategies (Choy et al., 2020).

One of the factors with the greatest weight in the maintenance of health is healthy lifestyle, which can be understood as a person's frequent behavior that favors the maintenance of good health and decreases the risks of morbidity and mortality (Vidal Gutiérrez et al., 2014). For the World Health Organization (WHO, 1999), maintaining a healthy lifestyle (HLS) is important because it reduces the risk of serious diseases, it is a way of living and enjoying more aspects of life, and it can benefit not only the person who implements it daily, but also his or her entire family by adopting this healthy lifestyle. An HLS may be associated with the presence of health-promoting behaviors, such as a healthy diet or systematic physical activity, or conversely, with the absence or avoidance of unhealthy behaviors, such as tobacco use, sedentary lifestyle, consumption of processed foods, etc. (WHO, 1999).

One of the factors that has been studied in relation to both the adoption and maintenance of lifestyles is the sense of coherence (SOC), a concept introduced by Antonovsky (1996). Originally, the concept referred to the ability to identify and use internal and external resources to facilitate the successful management of stressors and thus maintain and develop health; however, currently this variable has been studied as a protective factor for health in different types of populations, associated with health-related lifestyles.

There is evidence that SOC has a strong relationship with health behaviors through two pathways (Eriksson, 2006). On the one hand, it can behave as a protective factor against risk behaviors, where people with a strong SOC are less likely to consume tobacco and alcohol or be physically inactive (Escobar-Castellanos et al., 2019a,b; Wainwright et al., 2007) or it is associated, for example, with a lower presence of smoking, alcohol and substance use behaviors in young people (da-Silva-Domingues et al., 2022). On the other hand, it is positively associated with preventive health behaviors, such as having higher levels of physical activity and better eating habits (da-Silva-Domingues et al., 2022) or a higher level of physical exercise, consuming more fruits, vegetables and fiber regardless of age, social class and educational level (Escobar-Castellanos et al., 2019a,b; Wainwright et al., 2007) or having better glycemic control (Márquez-Palacios et al., 2021).

Some of the studies that reflect the relationship between SOC and HLS report that there is a difference between HLS profiles between women and men, the former with a profile more motivated in maintaining an HLS and in promoting it (Adorni et al., 2021; Suraj and Singh, 2011). Furthermore, this is linked to stress, since, by obtaining a high SOC, it could allow them to cope with stressful situations by inhibiting negative factors that could develop due to an HLS (Escobar-Castellanos et al., 2019a,b). The latter is important, given that migration has been found to have a stressful effect on people that can negatively affect physical, mental and emotional aspects of them. In a certain way, they have the hope of finding a new and better quality of life, facing stressful and unexpected situations such as loss and culture shock with their destination (Bonmatí-Tomas et al., 2019) or discrimination (Urzúa et al., 2020, 2021b).

Even though the importance of studying the factors involved in promoting the health and wellbeing of migrants in order to facilitate their adaptation and improve their quality of life in the new environment is well-known, it has not been possible to find research that addresses the relationship between this variables, which gives rise to the research question that underpinned this study on whether there is a relationship between the SOC and lifestyle in the migrant population.

The objective of this research, therefore, was to provide evidence on the relationship between the variables SOC and HLS in the migrant population. The working hypothesis is that the SOC is positively related to HLS in the migrant population, that is, the greater the SOC, the greater the adoption of healthy habits.

## 2 Method

This study was conducted under a non-experimental cross-sectional design, which only allows us to establish associations between the variables under study.

### 2.1 Participants

According to the National Institute of Statistics of Chile, in 2022 there were 1,615,074 foreigners living in Chile, of which 59% belonged to Venezuela, Peru and Colombia, that is, 967,429 people. Considering this number, with a confidence level of 95% and a margin of error of 5%, there should be a minimum of 385 participants. As an inclusion criterion, it was established that the participants had to have resided in Chile for a period of more than 3 months, since this is the time limit for the tourist visa in the country. Questionnaires were completed by 1,844 migrants between 18 and 82 years of age, regardless of their migratory status. A description of the sociodemographic characteristics of the participants can be found in Table 1.

**Table 1:** Sociodemographic characteristics of the participants.

**Variable**	**Category**	**Total (*n* = 1844), *n* (%)**
Sex	Man	918 (49.8)
	Woman	926 (50.2)
Nationality	Colombia	611 (33.1)
	Perú	608 (33)
	Venezuela	625 (33.9)
Years in Chile	< 5 years	1,132 (61.4)
	6–10 years	463 (25.1)
	>10 years	247 (13.4)
	No answer	2 (0.1)
Occupation	Employee	1,409 (76.4)
	Retired	22 (1.2)
	Unemployed	220 (11.9)
	Household worker	99 (5.4)
	Student	67 (3.6)
	No answer	27 (1.5)
Educational level	Incomplete primary education	61 (3.3)
	Primary education	233 (12.6)
	Secondary education	645 (35)
	Incomplete technical education	199 (10.8)
	Technical education	305 (16.5)
	Incomplete university education	131 (7.1)
	University education	230 (12.5)
	Postgraduate	36 (2)
	No answer	4 (0.2)
Income	< 125 US$	140 (7.6)
	126–375 US$	363 (19.7)
	376–750 US$	824 (44.7)
	751–1,250 US$	334 (18.1)
	1,251–1,875 US$	102 (5.5)
	>1,876 US$	67 (3.6)
	No answer	14 (0.8)
City	Arica	452 (24.5)
	Antofagasta	450 (24.4)
	Santiago	492 (26.7)
	Temuco	450 (24.4)

### 2.2 Procedures

This research was approved by the scientific ethics committee of the Universidad Católica del Norte. All ethical requirements included in the Helsinki declaration have been followed. It should be noted that each participant identified through this method signed a pre-participation consent form. In recognition of their collaboration, they were provided with a financial incentive equivalent to US$10. A non-representative sampling was carried out, trying to have a similar number of participants per country in each city, as well as a similar number of men and women for each national group. Participants were selected using a non-representative type of sampling, combining snowball and selective sampling, starting with a seed sample in commercial establishments and places frequently attended by migrants in various cities in Chile, such as the Jesuit Migrant Service, the Department of Foreigners and Migration, health centers, workplaces, and neighborhoods with a high number of migrant inhabitants (north, center and south of the country).

### 2.3 Measures

#### 2.3.1 Sense of coherence

We used the Life Orientation Scale - 13 (SOC-13), developed by Antonovsky (1993). This scale has reported good psychometric indicators in different Latin populations (Guerrero, 2018; Mafla et al., 2021; Márquez-Palacios et al., 2024; Saravia et al., 2014). The instrument consists of 13 items with responses on a seven-score Likert-type scale, ranging from always (1) to never (7). The questionnaire is divided into three subscales representing the three theoretical dimensions related to SOC:

- Comprehensibility: consists of five items and refers to the degree to which individuals make cognitive sense of present and future stimuli in their lives. It assesses the ability to make logical and orderly connections in their environment and to believe that life is predictable. Individuals with a high score on this subscale perceive stimuli as orderly, consistent, and predictable, allowing them to adapt more effectively to various situations.
- Manageability: includes four items and assesses the degree to which individuals believe they have access to the necessary resources to cope with the demands of the environment. It focuses on the availability of resources, whether under the control of the individual or others. This dimension is related to self-efficacy and competence but differs in that it refers to the perception that resources are available to the person. A high score on this subscale is associated with a positive view of life in general.
- Meaningfulness: this dimension is primarily emotional and motivational in nature. Consists of four items and refers to the value that the individual attaches to what happens in his or her life, regardless of how it occurs. It assesses the desire, emotions, and values associated with life events. A high score on this subscale indicates that the person finds meaning and purpose in life, considers challenges worth effort, and is satisfied with his or her life in general.

Given the unequal number of items that make up each factor, for statistical analysis and comparison purposes, the mean of each factor was used instead of the sum of the raw scores. In the present study, the reliability of the total scale, as measured by Cronbach's alpha, is 0.68.

#### 2.3.2 Lifestyles

The FANTASTICO instrument was used to evaluate this variable, which has been used for different population-based surveys. It has reported adequate psychometric properties (Díaz and Peña, 2022; Ramírez-Vélez and Agredo, 2012; Villar López et al., 2016). The instrument consists of 30 questions scored from 0 to 2, which make it possible to identify and measure the lifestyle of a particular population (Rodríguez-Moctezuma et al., 2003). This scale incorporates 10 factors, generating with the first 10 letters the acronym FANTASTICO: **F**amily and friends, **A**ssociativity & Physical and social activity, **N**utrition, **T**oxicity & smoking, **A**lcohol & other drugs, **S**leep & stress, **T**ype of Personality & school/work satisfaction, **I**ntrospection & Self-image, **C**ontrol of Health & sexuality management, **O**ther behaviors & Order. In the present study, the reliability of the total scale, as measured by Cronbach's alpha, is 0.85.

### 2.4 Data analysis

First, the sociodemographic characteristics of the study participants are described. Descriptive data for each of the scales are then presented. The mean differences by sex, age and country of origin in the scores of the SOC and FANTASTICO scales are then analyzed. Since some differences were observed for these variables, it was decided to include Sex, age, years in Chile, country of origin, income, educational level and city of residence as control variables in the following analysis. Next, a path analysis was performed to investigate the relationship between the dimensions of both scales. The dimensions of the SOC were included as predictor variables, the dimensions of the FANTASTICO scale as response variables. Model fit was evaluated using the root mean square error of approximation (RMSEA), the comparative fit index (CFI) and the Tuker-Lewis index (TLI). Sex, age, years in Chile, country of origin, income, educational level and city of residence were included as control variables. Analyses were performed with the statistical packages SPSS v.24 and Mplus v.8.9.

## 3 Results

The descriptive analyses of the scores of the SOC scale and the “FANTASTIC” scale and their respective dimensions can be seen in Table 2.

**Table 2:** Descriptives.

**Variables**	**Total**		**Venezuela**		**Perú**		**Colombia**	
	***M***	***SD***	***M***	***SD***	***M***	***SD***	***M***	***SD***
SOC	4.40	0.82	4.38	0.83	4.43	0.84	4.40	0.80
Meaningfulness	4.55	0.92	4.52	0.92	4.59	0.93	4.54	0.90
Manageability	4.23	0.99	4.20	0.99	4.27	0.99	4.23	0.97
Comprehensibility	4.42	1.09	4.41	1.09	4.43	1.08	4.42	1.1
LS	1.37	0.27	1.38	0.27	1.36	0.28	1,37	0.27
F	1.35	0.57	1.37	0.57	1,32	0.56	1.37	0.58
A1	0.87	0.54	0.88	0.54	0.85	0.52	0.89	0.55
N	1.17	0.38	1.17	0.39	1.18	0.36	1.17	0.37
T1	1.60	0.58	1.59	0.58	1.63	0.54	1.57	0.60
A2	1.71	0.34	1.71	0.31	1.73	0.36	1.68	0.34
S	1.26	0.59	1.27	0.58	1.21	0.62	1.31	0.55
T2	1.53	0.40	1.54	0.39	1.52	0.40	1.54	0.41
I	1.36	0.51	1.35	0.52	1.36	0.49	1.36	0.51
C	1.25	0.48	1.27	0.48	1.19	0.48	1.28	0.46
O	1.49	0.56	1.55	0.53	1.42	0.59	1.50	0.54

SOC, Sense of coherence; LS, lifestyle; F, family and friends; A1, physical and social activity/associativity; N, nutrition; T1, toxicity/smoking; A2, alcohol/other drugs; S, sleep and stress; T2, personality type and school/job satisfaction; I, Self-image/introspection; C, health and sexuality management; O, order/other behaviors.

In relation to the SOC, the factor with the highest mean was that of meaningfulness, both in the total sample and in the country sample. No statistically significant differences were found between the country means in any of the SOC variables.

SOC is found to be directly related to age both at the level of the total scale (*r* = 0.082; *p* = 0.000) and the subscales of manageability (*r* = 0.095; *p* = 0.000) and comprehensibility (*r* = 0.083; *p* = 0.001). When broken down by country, it is found that in the Colombian sample, SOC correlates directly with age in the total score (*r* = 0.089; *p* = 0.028) and manageability (*r* = 0.092; *p* = 0.023). In the Venezuelan sample, it correlates with total scale (*r* = 0.102; *p* = 0.011), comprehensibility (*r* = 0.118; *p* = 0.003) and manageability (*r* = 0.111; *p* = 0.006), while none of the SOC components is related to age in the Peruvian sample. No differences were found in the SOC given by sex.

Regarding lifestyle, the most prevalent lifestyle in the sample evaluated was low drug use, a behavior that was repeated in the three populations studied. The least prevalent lifestyle in the total sample and by country was physical activity. Statistically significant differences were observed in sleep and stress behaviors [*F* = 4.372_(2.1841);_ *p* = 0.013], where the mean of the Peruvian population evaluated is significantly lower than that of Colombia (*p* = 0.11), in health and sexuality control [*F* = 7.251_(2.1841);_ *p* = 0.001], where the Peruvian mean is significantly lower than that of Colombia (*p* = 0.001) and Venezuela (*p* = 0.009) and in the order variable [*F* = 8.619_(2.1841);_ *p* = 0.000], where the mean of the Peruvian population is significantly lower than that of Venezuela (*p* = 0.000). When analyzing differences by sex, different behavior is found in the variables physical activity and associativity [*t*_(1, 842, 1, 840.60)_ = 1.997; *p* = 0.046], where the mean of men (0.90) is higher than that of women (0.85), Tobacco [*t*_(1, 841, 1, 838.39)_ = −2.109; *p* = 0.035], where the mean of women (1.63) is greater than that of men (1.57), sleep and stress [*t*_(1, 842, 1, 841.86)_ = 2.473; *p* = 0.013], where the mean of men (1.30) is greater than that of women (1.23) and in introspection [*t*_(1, 841, 1, 838.54)_ = −4.145; *p* = 0.000], where the mean of men (1.41) is greater than that of women (1.31).

The correlation between the factors that make up both the lifestyle scale and the sense of coherence scale, stratified by country, can be seen in Tables 3–5.

**Table 3:** Correlations between variables (Colombians).

**Var**.	**SOC**	**MF**	**MB**	**CH**	**LS**	**F**	**A1**	**N**	**T1**	**A2**	**S**	**T2**	**I**	**C**	**O**
SOC	1	0.67^*^	0.86^*^	0.83^*^	0.37^*^	0.37^*^	−0.01 −0.05	0.17^*^	0.11^*^	0.24^*^	0.34^*^	0.33^*^	0.39^*^	0.18^*^	0.19
MF		1	0.33^*^	0.40^*^	0.26^*^	0.29^*^	0.04	0.19^*^	0.10^*^	0.16^*^	0.26^*^	0.22^*^	0.17^*^	0.18^*^	0.21^*^
MB			1	0.60^*^	0.31^*^	0.31^*^	−0.01	0.11^*^	0.07	0.18^*^	0.29^*^	0.26^*^	0.36^*^	0.16^*^	0.13^*^
CH				1	0.31^*^	0.28^*^		0.11^*^	0.10^*^	0.24^*^	0.27^*^	0.32^*^	0.38^*^	0.10^*^	0.11^*^
LS					1	0.61^*^	0.40^*^	0.61^*^	0.40^*^	0.51^*^	0.71^*^	0.66^*^	0.67^*^	0.67^*^	0.52^*^
F						1	0.12^*^	0.34^*^	0.19^*^	0.18^*^	0.42^*^	0.30^*^	0.40^*^	0.42^*^	0.44^*^
A1							1	0.28^*^	−0.07	−0.12^*^	0.28^*^	0.07	0.18^*^	0.29^*^	0.07
N								1	0.24^*^	0.22^*^	0.40^*^	0.33^*^	0.30^*^	0.38^*^	0.29^*^
T1									1	0.53^*^	−0.01	0.24^*^	0.14^*^	0.19^*^	0.14^*^
A2										1	0.07	0.42^*^	0.30^*^	0.18^*^	0.14^*^
S											1	0.53^*^	0.55^*^	0.48^*^	0.36^*^
T2												1	0.50^*^	0.26^*^	0.27^*^
I													1	0.31^*^	0.20^*^
C														1	0.38^*^
O															1

SOC, Sense of coherence; MF, meaningfulness; MB, manageability; CH, comprehensibility; LS, lifestyle; F, family and friends; A1, physical and social activity/associativity; N, nutrition; T1, toxicity/smoking; A2, alcohol/other drugs; S, sleep and stress; T2, personality type and school/job satisfaction; I, self-image/introspection; C, health and sexuality management; O, order/other behaviors. ^*^p < 0.05.

**Table 4:** Correlations between variables (Peruvians).

**Var**.	**SOC**	**MF**	**MB**	**CH**	**LS**	**F**	**A1**	**N**	**T1**	**A2**	**S**	**T2**	**I**	**C**	**O**
SOC	1	0.78^*^	0.87^*^	0.82^*^	0.39^*^	0.40^*^	−0.00 −0.05	0.17^*^	0.12^*^	0.22^*^	0.35^*^	0.37^*^	0.38^*^	0.21^*^	0.22^*^
MF		1	0.50^*^	0.53^*^	0.30^*^	0.32^*^	0.06 −0.03	0.18^*^	0.07	0.16^*^	0.31^*^	0.27^*^	0.23^*^	0.20^*^	0.20^*^
MB			1	0.58^*^	0.38^*^	0.37^*^		0.16^*^	0.11^*^	0.20^*^	0.32^*^	0.34^*^	0.36^*^	0.20^*^	0.20^*^
CH				1	0.28^*^	0.30^*^		0.09^*^	0.12^*^	0.20^*^	0.23^*^	0.29^*^	0.33^*^	0.11^*^	0.14^*^
LS					1	0.61^*^	0.41^*^	0.56^*^	0.41^*^	0.54^*^	0.72^*^	0.68^*^	0.67^*^	0.65^*^	0.54^*^
F						1	0.19^*^	0.27^*^	0.17^*^	0.17^*^	0.44^*^	0.31^*^	0.33^*^	0.41^*^	0.47^*^
A1							1	0.27^*^	−0.03	−0.05	0.26^*^	0.10^*^	0.16^*^	0.24^*^	0.10^*^
N								1	0.20^*^	0.22^*^	0.38^*^	0.33^*^	0.29^*^	0.28^*^	0.26^*^
T1									1	0.54^*^	0.03	0.25^*^	0.18^*^	0.18^*^	0.11^*^
A2										1	0.09^*^	0.44^*^	0.41^*^	0.16^*^	0.13^*^
S											1	0.52^*^	0.48^*^	0.48^*^	0.41^*^
T2												1	0.53^*^	0.29^*^	0.24^*^
I													1	0.35^*^	0.19^*^
C														1	0.43^*^
O															1

SOC, Sense of coherence; MF, meaningfulness; MB, manageability; CH, comprehensibility; LS, lifestyle; F, family and friends; A1, physical and social activity/associativity; N, nutrition; T1, toxicity/smoking; A2, alcohol/other drugs; S, sleep and stress; T2, personality type and school/job satisfaction; I, self-image/introspection; C, health and sexuality management; O, order/other behaviors. ^*^p < 0.05.

**Table 5:** Correlations between variables (Venezuelans).

**Var**.	**SOC**	**MF**	**MB**	**CH**	**LS**	**F**	**A1**	**N**	**T1**	**A2**	**S**	**T2**	**I**	**C**	**O**
SOC	1	0.71^*^	0.88^*^	0.83^*^	0.43^*^	0.37^*^	0.02	0.25^*^	0.12^*^	0.26^*^	0.38^*^	0.40^*^	0.44^*^	0.22^*^	0.19^*^
MF		1	0.42^*^	0.41^*^	0.35^*^	0.27^*^	−0.01	0.27^*^	0.14^*^	0.18^*^	0.35^*^	0.32^*^	0.30^*^	0.20^*^	0.14^*^
MB			1	0.63^*^	0.38^*^	0.32^*^	0.06	0.20^*^	0.09^*^	0.22^*^	0.32^*^	0.33^*^	0.40^*^	0.18^*^	0.20^*^
CH				1	0.32^*^	0.31^*^	−0.01	0.15^*^	0.08^*^	0.23^*^	0.27^*^	0.33^*^	0.35^*^	0.17^*^	0.11^*^
LS					1	0.59^*^	0.42^*^	0.64^*^	0.37^*^	0.48^*^	0.72^*^	0.66^*^	0.68^*^	0.64^*^	0.52^*^
F						1	0.07	0.26^*^	0.13^*^	0.18^*^	0.48^*^	0.33^*^	0.38^*^	0.41^*^	0.38^*^
A1							1	0.28^*^	−0.07	−0.08^*^	0.29^*^	0.09^*^	0.22^*^	0.30^*^	0.07^*^
N								1	0.21^*^	0.28^*^	0.45^*^	0.39^*^	0.36^*^	0.35^*^	0.28^*^
T1									1	0.52^*^	−0.03	0.22^*^	0.14^*^	0.18^*^	0.15^*^
A2										1	0.07	0.41^*^	0.28^*^	0.11^*^	0.13^*^
S											1	0.50^*^	0.54^*^	0.41^*^	0.33^*^
T2												1	0.52^*^	0.26^*^	0.26^*^
I													1	0.30^*^	0.20^*^
C														1	0.40^*^
O															1

SOC, Sense of coherence; MF, meaningfulness; MB, manageability; CH, comprehensibility; LS, lifestyle; F, family and friends; A1, physical and social activity/associativity; N, nutrition; T1, toxicity/smoking; A2, alcohol/other drugs; S, sleep and stress; T2, personality type and school/job satisfaction; I, self-image/introspection; C, health and sexuality management; O, order/other behaviors. ^*^p < 0.05.

The results of the path analysis showed that the model presented excellent goodness of fit (Parameters = 165; RMSEA = 0.00; CFI = 1; TLI = 1), therefore, a good representation of the observed relationships.

Significance was positively related of small magnitude (*r* > 0.20; Cohen, 1988) with the nutrition dimension (β = 0.210, *p* = 0.000), and of slight magnitude (*r* > 0.10; Cohen, 1988) with the dimension's family and friends (β = 0.159, *p* = 0.000), smoking (β = 0.140, *p* = 0.000), alcohol and other drugs (β = 0.157, *p* = 0.000), sleep and stress (β = 0.180, *p* = 0.000), personality type (β = 0.175, *p* = 0.000), introspection (β = 0.059, *p* = 0.017), health control (β = 0.141, *p* = 0.000), and order (β = 0.141, *p* = 0.000). While negatively with physical activity (β = −0.113, *p* = 0.000).

In the case of the manageability dimension, this was positively related and of small magnitude (*r* > 0.20; Cohen, 1988) with the dimension's family and friends (β = 0.203, *p* = 0.000) and introspection (β = 0.234, *p* = 0.000), while of slight magnitude, with physical activity (β = 0.110, *p* = 0.000), nutrition (β = 0.089, *p* = 0.002), alcohol and other drugs (β = 0.106, *p* = 0.000), sleep and stress (β = 0.181, *p* = 0.000), personality type (β = 0.168, *p* = 0.000), health control (β = 0.118, *p* = 0.000), and orderliness (β = 0.132, *p* = 0.000). Manageability did not present statistically significant relationships with the tobacco dimension (β = 0.051, *p* = 0.068).

Finally, the comprehensibility dimension, was positively and of slight magnitude related with the dimension's family and friends (β = 0.106, *p* = 0.000) alcohol and other drugs (β = 0.128, *p* = 0.000), personality type (β = 0.151, *p* = 0.000), and introspection (β = 0.191, *p* = 0.000). While negatively with physical activity (β = −0.060, *p* = 0.042). Comprehensibility did not present statistically significant relationships with the dimension's nutrition (β = −0.021, *p* = 0.475), smoking (β = 0.043, *p* = 0.127), sleep and stress (β = 0.053, *p* = 0.057), health control (β = −0.006, *p* = 0.837), and order (β = −0.023, *p* = 0.432).

**Figure 1:**
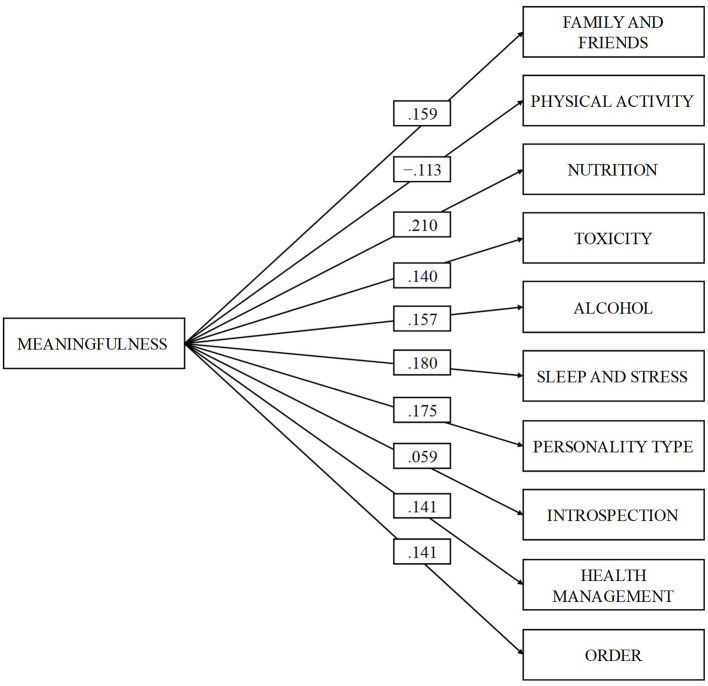
Correlation between the meaningfulness and the dimensions of the “fantastic” scale. All relationships were statistically significant (*p* < 0.05).

**Figure 2:**
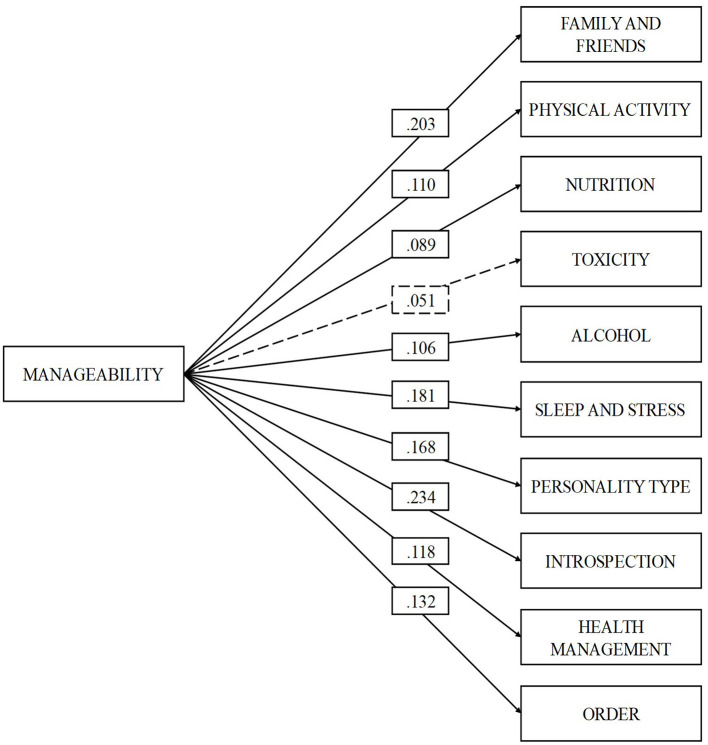
Correlation between the manageability and the dimensions of the “fantastic” scale. Statistically significant relationships (*p* < 0.05) are represented by solid lines, while non-significant relationships (*p* < 0.05) are represented by dashed lines.

**Figure 3:**
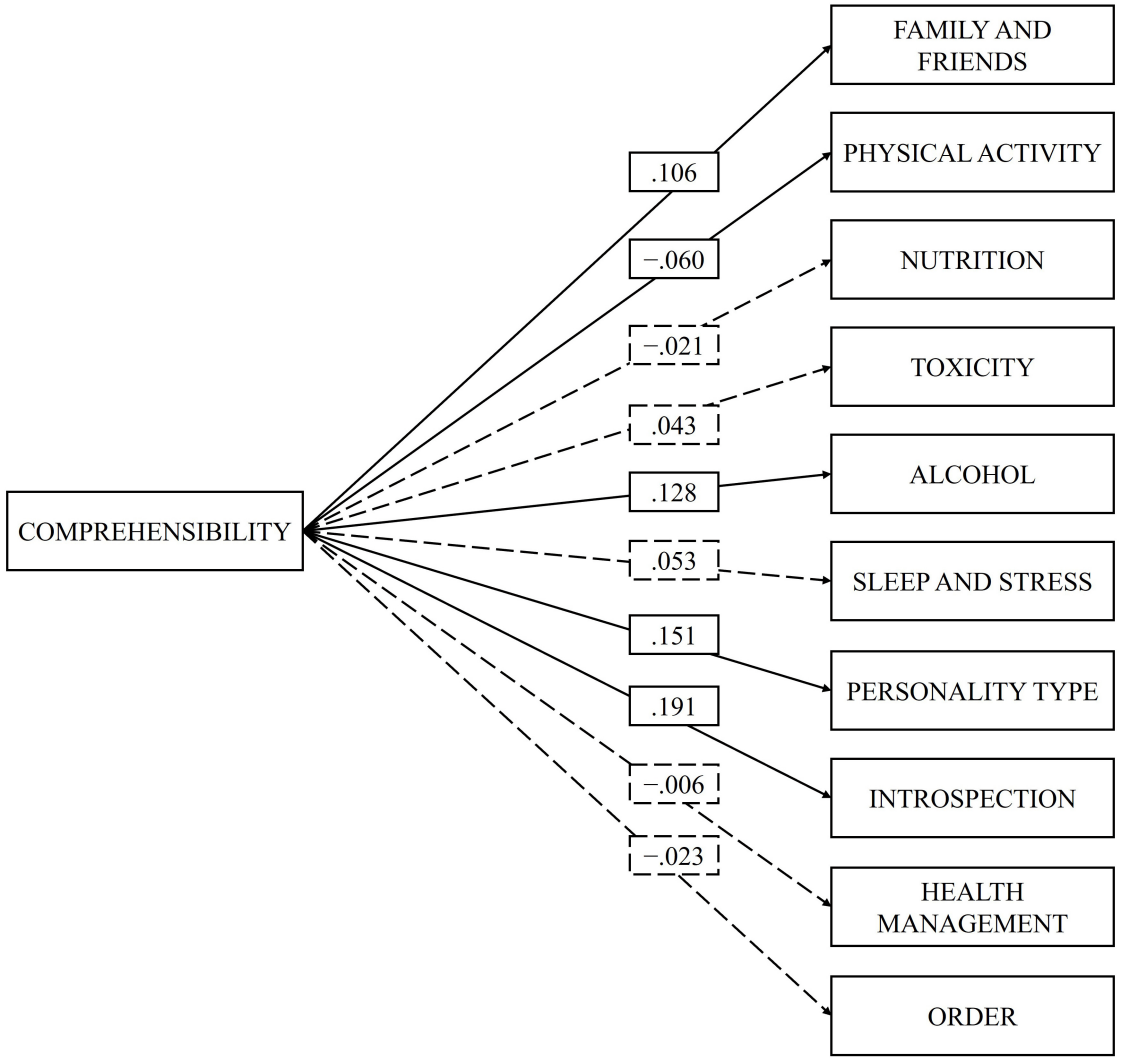
Correlation between the comprehensibility and the dimensions of the “fantastic” scale. Statistically significant relationships (*p* < 0.05) are represented by solid lines, while non-significant relationships (*p* < 0.05) are represented by dashed lines.

In Figures 1–3, the relationships between the dimensions of SOC and the dimensions of “fantastic” can be observed. The model is presented in three separate figures for each dimension of the SOC for better understanding. This does not mean that the model has been tested three different times.

## 4 Discussion

The research that has been carried out was aimed at analyzing the relationship between the SOC and healthy lifestyle in a migrant population. Regarding the factors that make up the SOC, the highest mean was that of meaningfulness, which could imply that the people evaluated have a great sense of meaning and purpose in life and can consider that the challenges presented upon arrival are worth the effort, being satisfied with their life in general.

When analyzing the relationship between SOC and age, the results are not conclusive, given that the associations will vary depending on the country, and even no association is found, as is the case of the Peruvian population. In the samples where relationships are observed, these are found mainly with the comprehensibility and manageability dimension, which seems to be congruent with similar research where it is reported that as people age, they can comprehensively understand their environment and their life experiences and perceive that they have greater resources available, having a more positive view of life in general. In this sense, it has been reported that individuals over the age of 60 appeared to have higher scores in the SOC (Fernández-Martínez et al., 2017). This would imply that the older migrant population could have a higher capacity to establish logical connections, and it is also hypothesized that the length of residence of migrants in Chile could be an influential factor, which would allow them to adapt better to stressful situations in their lives. Despite this, some researchers have also reported contrary evidence, showing an inverse relationship between SOC and age, suggesting that the SOC deteriorates over time (Burger et al., 2014), coinciding with what was suggested by Antonovsky (1987), who mentions that the SOC increases with age up to 30 years, then decreases over time. Unfortunately, we have not found other studies in a similar population to contrast this finding. Despite this, it is logical to hypothesize that older age would imply the development of cognitive skills of an integrative type, which would allow a greater understanding of the environment, as well as the perception of a greater sense of it, especially when the decision has been made to live in a different country to which the person is integrating as time goes by.

In relation to healthy lifestyle, the variable alcohol/other drugs and toxic/tobacco turned out to be an important protective element in their daily lives (low presence of this factor). Possibly, this behavior, or the low frequency of it, is mainly associated with the focus of the migrating population on their purpose of building a better life than the one they had in their country of origin, avoiding unhealthy behaviors that may hinder this goal, such as drug use. It is worth mentioning that the population that participated in this research corresponds to first-generation migrants, most of whom have been in Chile for < 5 years, so their behavioral patterns are mainly oriented toward settling in Chile and achieving their migratory objectives. This is consistent with the fact that we have also found that the least settled lifestyle is physical and social activity and associativity, which must be related to the low capacity to have time for activities of this type, considering as a priority activities that involve monetary retribution over leisure activities. It is worth mentioning that these lifestyle decisions directly influence the health and wellbeing of the person, as mentioned by authors who studied mental health, wellbeing and quality of life in the Peruvian and Colombian population in Chile, where they show that migration has an impact on the physical health, mental health and wellbeing of the migrant population, which makes attention to these aspects essential, especially in a migratory context where changes in the environment and adaptation to a new reality can have a significant impact on health (Urzúa and Cabieses, 2018). Although the current research on the relationship between the duration of residence in the host country and the presence of risk behaviors such as smoking, physical inactivity, drug use, unhealthy eating, is not conclusive and seems to be highly context dependent (Juárez et al., 2022), we believe it is important to continue exploring the effect of this variable, but incorporating the effect of third variables, such as the SOC, under a longitudinal design.

With respect to the results by sex, men present greater protective behaviors compared to women, with higher means in almost all the dimensions, even though these differences are only statistically significant in three of them: physical activity and associativity, sleep and stress, and the internal image/introspection variable, where men would have greater protective behaviors compared to women. What may influence the adoption of less protective behaviors for women's health are gender roles, where social pressure and traditional beliefs lead women to assume family caregiver roles, as well as child-rearing and housework responsibilities. This role burden has a negative impact on their physical and mental health, limiting women's ability to engage in personal activities and manage their time, as evidenced in the study (Fonseca-Mardones, 2020). Even though it is a different population, this differential behavior in lifestyles has also been reported in the university population, where it was found that women generally have a lower use of tobacco, illicit substances and alcohol and consume more fruits and vegetables (Braga et al., 2023). On the other hand, men had a higher level of physical activity, consume less sweets and had a more restful sleep (El Ansari et al., 2011). Our findings differ from those found in the Italian population (El Ansari et al., 2011), where it was found that women had a higher level of motivation to maintain a healthy lifestyle and encourage this practice in their environment, and with those reported by Alonso-Castillo et al. (2018), who reported that men had a higher harmful alcohol consumption than women in the university population.

When the SOC and lifestyle were analyzed, it was found that, of the three components of SOC, the one that seems to have the strongest association with lifestyles is meaningfulness, unlike, for example, what was reported among university students, where the strongest relationship with lifestyles was found in the comprehensibility dimension (Escobar-Castellanos et al., 2019b). This is logical in the population studied, since it would imply that the more meaning a migrant person finds in his or her life (actions, consequences, meaning), the greater the probability that he or she will have healthy lifestyles, which are probably perceived as requirements to perform successfully and fulfill the purposes for which he or she has migrated and which give meaning to his or her existence. What is striking is that the physical and social activity variable correlates inversely with the significance variable (as well as with comprehensibility). One possible explanation is suggested by Ambiado Cortes et al. (2022), who suggest that excessive working hours are a common problem among immigrants in Chile, whether they work part-time in several jobs or full-time. This would imply that as immigrants integrate into the labor market and urban life, their physical activity tends to decrease. This reduction could negatively affect their individual and social wellbeing. On the other hand, it was found that the variable family and friends correlated directly with all SOC variables, providing evidence between the association of this with having a support network.

Although the present study may be of great relevance for the implementation of interventions aimed at strengthening the health of the migrating population, it is important to point out some limitations of these results. First, the present study is of cross-sectional design, so it is only possible to visualize associations between the variables under study, without the possibility of attributing causal effects of one over the other. Secondly, being a non-probabilistic sample, and even though we have tried to include an equal number of participants per country and sex, the sample may be biased toward immigrants who wanted to participate in the study and the conclusions could not be extended to migrants from other countries not considered in this study, so that future research could reproduce these analyses in migrants from other countries, as well as in different host countries.

## 5 Conclusion

The health of a person is determined by multiple variables, being the presence of healthy behavioral patterns, known as lifestyles, one of the most important factors in maintaining good health. There is little research that relates a greater presence of these healthy lifestyles with other variables that may influence the acquisition and/or maintenance of these behaviors. In this research, we provide evidence of the close relationship between the presence of a high sense of coherence with healthy lifestyles in the migrant population, a relationship that is maintained controlling for variables such as sex, age, years living in the country, country of origin, income, educational level and city of residence.

We believe it is relevant to highlight that although there is little evidence in this regard, given that we have only been able to find in the literature an experimental study in people at risk of type 2 diabetes (Nilsen et al., 2015), it would seem that the intentional modification of SOC can be a good predictor of changes in lifestyles, since it would allow people to realize the impact that their behaviors can have on the management of their health. These results provide evidence of the need to consider variables such as manageability, meaningfulness and comprehensibility in the implementation of field interventions in the migrant population, since they constitute real coping strategies that will strengthen the health of migrants.

## Data Availability

The raw data supporting the conclusions of this article will be made available by the authors, without undue reservation.
